# Supplementary material for: Narrative–affect discrepancy as a regulated degree of freedom in 351,734 relationship narratives
Source: PLoS One. 2026 May 12;21(5):e0348715. doi: 10.1371/journal.pone.0348715 (PMC13166951; doi:10.1371/journal.pone.0348715)
Supplement: S1 Text — Robustness of convex-hull area contraction under axis-wise rescaling and quantile threshold perturbation (±5 percentile shifts) of the clipped space. (PDF) [file pone.0348715.s001.pdf]

S1 Text. Sensitivity analysis of expressive area

This section reports robustness checks for expressive-area estimates under axis-wise rescaling of the clipped (N', A') geometry and under perturbation of quantile thresholds used for regime labeling. Values are computed by the same exported analysis pipeline used for the main text and are reported directly from Source Data.

Table 1: *

Table S1: Sensitivity of expressive-area contraction under axis-wise rescaling in the (N', A') plane.

Scaling condition	Human area	LLM area	Contraction ratio
Baseline ($\lambda = 1/\text{SD}$)	13.27	7.80	1.70
N-heavy ($\lambda_N \times 1.5$)	19.90	11.70	1.70
A-heavy ($\lambda_A \times 1.5$)	19.90	11.70	1.70
Uniform weights ($\lambda = 1$)	13.27	7.80	1.70

Table 2: *

Table S2: Sensitivity of regime prevalence to quantile threshold perturbation. Contraction ratio (human/LLM hull area) is stable across conditions.

Threshold condition	Coupled (%)	Under. (%)	Over. (%)	Collapse (%)	Ratio
Baseline (75/75/25)	91.33	5.75	0.63	2.29	1.70
Relaxed (70/70/30)	86.33	8.51	0.72	4.44	1.70
Strict (80/80/20)	95.14	3.37	0.57	0.92	1.70

The contraction ratio is invariant to both rescaling and threshold perturbation, confirming that the geometric comparison is robust to reasonable analytic choices. Regime prevalence varies with threshold stringency as expected, but the four-regime qualitative structure is preserved across all conditions tested.
