# Supplementary material for: Narrative–affect discrepancy as a regulated degree of freedom in 351,734 relationship narratives
Source: PLoS One. 2026 May 12;21(5):e0348715. doi: 10.1371/journal.pone.0348715 (PMC13166951; doi:10.1371/journal.pone.0348715)
Supplement: S2 Text — Histogram-based density projections for (N′,A′), (N′,D′), and (A′,D′) from the clipped human corpus (Source Data: fig1_density_*.dat). (PDF) [file pone.0348715.s002.pdf]

S2 Text. Density projections of the clipped NCS

Two-dimensional density projections are rendered from exported histogram Source Data files (`fig1_density_NA.dat`, `fig1_density_ND.dat`, `fig1_density_AD.dat`). Colors encode $\ln(\rho + 1)$, where ρ is bin occupancy.

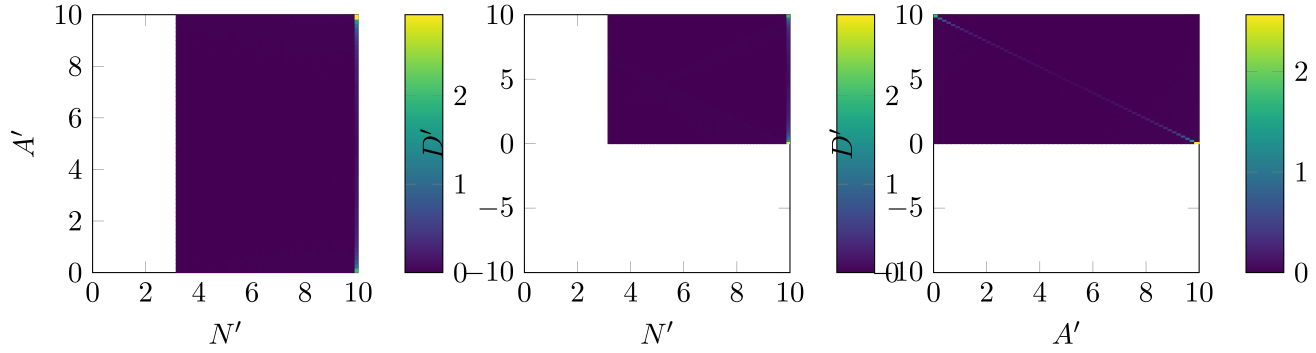

(a) N'–A' (b) N'–D' (c) A'–D'

Figure S1: Density projections of the clipped human NCS geometry.
