# Supplementary material for: Narrative–affect discrepancy as a regulated degree of freedom in 351,734 relationship narratives
Source: PLoS One. 2026 May 12;21(5):e0348715. doi: 10.1371/journal.pone.0348715 (PMC13166951; doi:10.1371/journal.pone.0348715)
Supplement: S3 Text — Descriptive statistics for humans and the aligned model used throughout regime and geometry analyses. (PDF) [file pone.0348715.s003.pdf]

S3 Text. Summary statistics

Descriptive statistics for humans and the aligned model in the clipped NCS.

Table 1: *

Table S3: Summary statistics for human and LLM trajectories in the clipped NCS.

Agent	Variable	Mean	SD	Min	Max
Human	N	2.43	1.80	0.00	10.00
Human	A	6.50	4.15	0.00	10.00
Human	$\|D\|$	5.34	2.89	0.00	10.00
LLM	N	1.69	1.31	0.00	7.59
LLM	A	4.05	2.52	0.64	9.38
LLM	$\|D\|$	2.82	2.37	0.00	8.37
