# Supplementary material for: Narrative–affect discrepancy as a regulated degree of freedom in 351,734 relationship narratives
Source: PLoS One. 2026 May 12;21(5):e0348715. doi: 10.1371/journal.pone.0348715 (PMC13166951; doi:10.1371/journal.pone.0348715)
Supplement: S4 Text — Pearson correlations among (N,A,D) in the clipped human corpus. (PDF) [file pone.0348715.s004.pdf]

S4 Text. Correlation structure

Pearson correlations among (N, A, D) are computed on the clipped human corpus. Note that D is defined as $N - A$ prior to clipping; thus the A–D association is mechanically constrained and is reported for completeness.

Table 1: *
Table S4: Pearson correlation matrix for (N, A, D) in the clipped human corpus.

	N	A	D
N	1.000	0.009	−0.008
A	0.009	1.000	−0.971
D	−0.008	−0.971	1.000
