# Supplementary material for: Narrative–affect discrepancy as a regulated degree of freedom in 351,734 relationship narratives
Source: PLoS One. 2026 May 12;21(5):e0348715. doi: 10.1371/journal.pone.0348715 (PMC13166951; doi:10.1371/journal.pone.0348715)
Supplement: S5 Text — Regime-level prevalence and mean locations rendered directly from exported Source Data. (PDF) [file pone.0348715.s005.pdf]

S5 Text. Regime-level summaries

Regime-level means and counts are reported directly from exported Source Data (table3_regime_stats.csv).

Table 1: *

Table S5: Regime-level summary statistics and prevalence in the NCS.

Regime	$\overline{N}$	$\overline{A}$	$\overline{D}$	Count	Prevalence (%)
Coupled	2.478	6.236	−3.758	321 248	91.33
Understatement	1.678	9.960	−8.283	20 223	5.75
Overstatement	9.415	0.186	9.228	2 223	0.63
Collapse	0.866	9.952	−9.087	8 040	2.29
