# Supplementary material for: Narrative–affect discrepancy as a regulated degree of freedom in 351,734 relationship narratives
Source: PLoS One. 2026 May 12;21(5):e0348715. doi: 10.1371/journal.pone.0348715 (PMC13166951; doi:10.1371/journal.pone.0348715)
Supplement: S6 Text — Bootstrap and permutation-test reporting for the area contraction comparison (Source Data: fig4_volume_compare.dat). (PDF) [file pone.0348715.s006.pdf]

S6 Text. Inference for expressive-area contraction

Uncertainty and significance reporting for the area contraction comparison. Because $D = N - A$ is a deterministic function of the two primary axes, three-dimensional hull volume is structurally zero on the exact data plane and any non-zero volume in the clipped (N', A', D') space reflects axis-wise clipping artifacts rather than genuine distributional spread. We therefore report hull area in the (N', A') plane. Values are reproduced from the exported analysis output (Source Data: `fig4_volume_compare.dat`).

Table 1: *
Table S6: Uncertainty and significance summary for expressive-area contraction in the clipped (N', A') plane.

Metric	Value
Human convex-hull area (bootstrap mean; $B = 100$, $n = 1{,}000$)	99.514
Human convex-hull area 95% CI	[98.596, 99.755]
LLM convex-hull area ($n = 1{,}000$)	58.679
Contraction ratio (Human / LLM)	1.696
Contraction ratio 95% CI	[1.680, 1.700]
Permutation test (two-sided; $T = 10{,}000$ shuffles)	$p < 0.0001$
Occupancy entropy, human (2D, 24×24 bins)	4.654
Occupancy entropy, LLM (2D, 24×24 bins)	5.103
