# Supplementary material for: Narrative–affect discrepancy as a regulated degree of freedom in 351,734 relationship narratives
Source: PLoS One. 2026 May 12;21(5):e0348715. doi: 10.1371/journal.pone.0348715 (PMC13166951; doi:10.1371/journal.pone.0348715)
Supplement: S7 Text — Fixed prompt template and decoding configuration used to generate the 1,000 model trajectories (llm_prompt_set.txt; llm-config.json). (PDF) [file pone.0348715.s007.pdf]

S7 Text. LLM prompt template and decoding

The fixed prompt template (`llm_prompt_set.txt`) and decoding configuration (`llm_config.json`) used to generate `nadi_llm_trajectories.parquet` are provided in the Supplementary Software. Provider/model identifier (or closest available tag), generation date range, and all decoding parameters (including any nucleus sampling, maximum tokens, and stopping criteria) are recorded in `llm_config.json`.
