# Supplementary material for: Narrative–affect discrepancy as a regulated degree of freedom in 351,734 relationship narratives
Source: PLoS One. 2026 May 12;21(5):e0348715. doi: 10.1371/journal.pone.0348715 (PMC13166951; doi:10.1371/journal.pone.0348715)
Supplement: Attachment — Submitted filename: PONE-D-26-02582_R1_Response Letter.pdf [file pone.0348715.s010.pdf]

Response to Reviewers

Manuscript ID: PONE-D-26-02582

Narrative–Affect Discrepancy as a Regulated Degree of Freedom in 351,734 Relationship Narratives

Ryan SangBaek Kim
Ryan Research Institute (RRI), Paris, France

27 March 2026

Dear Editor and Reviewers,

Thank you for the careful and constructive evaluation of this manuscript. I have revised the manuscript extensively in response to the review process, with particular attention to interpretive precision, statistical transparency, and reproducibility. The revision has substantially improved the manuscript's conceptual clarity, geometric validity, and uncertainty reporting.

Below I address the Journal Requirements first, followed by Reviewer #1 (including the attached detailed review report, `Review Comments.docx`), Reviewer #2 (including the attached question file, `CommentsPLOS.docx`), and Reviewer #3. Reviewer comments are shown in blue, responses in black, and summaries of manuscript changes in green. Page references correspond to the revised clean manuscript unless otherwise noted.

Three author-initiated revisions affect multiple sections and are therefore noted here once for transparency.

(i) **Geometric correction from three-dimensional hull volume to two-dimensional hull area.** During revision, I re-examined the geometry of the expressive-space analysis and identified a structural issue in the original submission. Because signed discrepancy is defined as $D = N - A$, the triplet (N, A, D) lies on a plane in three-dimensional space prior to clipping. The three-dimensional convex-hull volume originally reported therefore reflected an artifact introduced by separate axis-wise clipping in transformed coordinates rather than genuine distributional spread. In the revised manuscript, all geometric extent measures are computed as convex-hull area in the clipped (N', A') plane. Under this corrected procedure, the aligned language model occupies a $1.70\times$ smaller region than the human comparison baseline (bootstrap 95% CI $[1.68, 1.70]$; permutation $p < 0.0001$).

(ii) **Expanded statistical and uncertainty reporting.** The revised manuscript now reports the near-zero Pearson correlation between narrative complexity and affective intensity directly in the main text ($r = 0.009$), adds bootstrap confidence intervals for human hull area and the contraction ratio, reports a two-sided permutation test, and provides occupancy entropy estimates for both humans and the aligned language model. Supplementary sensitivity analyses now also make explicit that the four-regime structure and contraction ratio remain stable under quantile-threshold perturbation.

(iii) **Terminology and reproducibility harmonization.** The framework name is now used consistently as *Narrative–Control Space (NCS)* throughout. The earlier variant "NADI control space" has been removed. The revised manuscript also points more explicitly to the prompt template, decoding configuration, source-data files, and software bundle that underwrite the analyses.

Response to Journal Requirements

Journal Requirement: *Please ensure that your manuscript meets PLOS ONE's style requirements, including those for file naming.*

Response: This requirement has been addressed. All files have been renamed according to PLOS ONE submission conventions, and the manuscript, supplementary materials, and response letter have been checked for consistency with PLOS ONE style requirements. Section labels, figure and table references, and Supporting Information labels have been standardized throughout.

Change: Files renamed in PLOS ONE format. Supporting Information labels standardized throughout the submission.

Journal Requirement: *Please note that PLOS ONE has specific guidelines on code sharing for submissions in which author-generated code underpins the findings in the manuscript.*

Response: This requirement has also been addressed. The revised manuscript now includes a Code Availability statement specifying that the analysis code for preprocessing, density estimation, cost-landscape evaluation, and expressive-area/entropy computation is provided as Supplementary Software. The submission includes the scripts used to generate the reported source-data files as well as the prompt template and configuration materials needed to reproduce the aligned-model analysis.

Change: Code Availability statement added on p. 19. Supplementary Software bundle included and referenced in the manuscript and Supporting Information.

Journal Requirement: *If the reviewer comments include a recommendation to cite specific previously published works, please review and evaluate these publications to determine whether they are relevant and should be cited.*

Response: All such suggestions were evaluated for direct relevance to the present manuscript. Only works that materially strengthened the revised framing, methodological positioning, or interpretation were added. The bibliography was also updated to reflect the current dataset article and the related conceptual and methodological references cited in the revised Discussion.

Change: Reference list reviewed and updated for relevance and completeness. Data Availability statement updated on p. 19.

Response to Reviewer #1

Inline Comments

Comment: *The manuscript would benefit from clearer transitions between sections and deeper analysis in certain areas.*

Response: I agree that the original version could be improved in this regard. The Introduction, Methods, Results, and Discussion have been revised for stronger argumentative continuity. Several sections that were previously more descriptive now provide clearer interpretation, especially the global geometry analysis, the human-versus-LLM comparison, and the discussion of what is meant by "regulated" in the present paper.

Change: Introduction revised for continuity and concision on pp. 2–5. Results 3.1–3.5 clarified and expanded on pp. 11–15. Discussion 4.1–4.3 revised for interpretive precision on pp. 15–17.

Comment: *Proofread for grammar and ensure consistent formatting throughout.*

Response: The manuscript has been proofread throughout. Terminology, section titles, table references, and Supporting Information labels were standardized. In particular, the framework name is now consistently given as Narrative–Control Space (NCS), and outdated variants have been removed.

Change: Terminology standardized throughout the manuscript. Supporting Information labels and cross-references corrected on pp. 19–20 and in the Supplementary Information.

Comment: *The conclusion should be strengthened with more impactful statements.*

Response: The Conclusion has been substantially revised. It now foregrounds the manuscript's three main empirical findings: near-independence between N and A ($r = 0.009$), persistence of the four expressive regimes under threshold perturbation, and a human-to-LLM expressive-area contraction ratio of $1.70\times$ with robust inferential support. It then summarizes the implications for clinical interpretation, emotion-AI benchmarking, and alignment auditing.

Change: Conclusion revised in Section 4.5 on pp. 18–19.

Attached Review: `Review Comments.docx`

Reviewer #1 submitted a detailed attached review raising four major comments and four minor comments. I address them in order below.

Attached Review: *Major Comment 1. The use of "regulated" may be misleading unless the authors clarify whether it refers to active individual control or population-level statistical constraint.*

Response: I agree that this distinction needed to be made more explicit. The revised manuscript now clarifies in both the Introduction and the Discussion that the term "regulated" is used primarily in a descriptive, population-level sense. It refers to stable occupancy patterns and constraint structure in aggregate narrative data. The manuscript does not infer individual conscious

self-regulation directly from these distributions. A more mechanistic interpretation is discussed only as a future hypothesis for longitudinal or experimental work.

Change: Introduction revised to clarify the descriptive, population-level meaning of "regulated" on p. 4. Discussion 4.1 revised to restate the distinction between aggregate structure and individual-level mechanism on p. 15.

Attached Review: *Major Comment 2. The cost function J requires stronger justification. Why these terms, and why these weights?*

Response: Section 2.6 has been expanded to address this concern directly. The manuscript now explains that the quadratic penalties were chosen for parsimony and analytic tractability, that the discrepancy term is centered on the empirical median D^* rather than zero because the population does not converge on zero discrepancy, and that the weighting scheme is anchored in empirical dispersion. Most importantly, the revised text now states explicitly that J is an abductive, illustrative function rather than a fitted latent objective. Its purpose is to summarize the observed constraint structure in a transparent and reproducible way, not to claim recovery of individual optimization dynamics.

Change: Methods 2.6 expanded with a detailed justification paragraph on pp. 9–10.

Attached Review: *Major Comment 3. The quantile-based regime thresholds are data-driven and potentially arbitrary. The terminology surrounding "collapse" should also be clarified.*

Response: This concern has been incorporated in two ways. First, the revised manuscript now states explicitly that regime boundaries are quantile-based and data-driven, and that the coupled regime is defined as the complement of the three more extreme patterns rather than as a privileged equilibrium state. Second, supplementary sensitivity analyses show that the four-regime structure and the contraction ratio remain stable under reasonable threshold perturbations. The manuscript retains the label "collapse," but now defines it more carefully as a descriptive structural term for high affect with minimal narrative scaffolding, without implying clinical pathology.

Change: Methods 2.5 revised to clarify the data-driven nature of regime boundaries and to justify the use of the label "collapse" on p. 8. Supplementary threshold-perturbation sensitivity results added in S1 Text on p. 1 of the Supplementary Information.

Attached Review: *Major Comment 4. The interpretation of the LLM comparison risks over-attribution to alignment objectives. Alternative explanations should be acknowledged more directly.*

Response: I agree, and the revised manuscript now states this more explicitly. The Discussion acknowledges that the observed contraction may reflect multiple factors, including prompt design, decoding constraints, safety filtering, training distribution, and model architecture, and should not be attributed solely to alignment objectives. The aligned-model comparison is therefore framed as a descriptive contrast under a fixed prompt and decoding configuration rather than as a causal decomposition of alignment effects.

Change: Introduction revised to specify the fixed prompt and decoding context on p. 3. Discussion 4.3 and Limitations 4.4 expanded on pp. 17–18.

Attached Review: *Minor Comment 1. Terminology should be standardized.*

Response: This has been done. Narrative–Control Space (NCS) is now defined once and used consistently throughout. The earlier variant "NADI control space" has been removed.

Change: Terminology standardized throughout the manuscript and supplementary materials, including the Introduction on pp. 3–4.

Attached Review: *Minor Comment 2. The term "collapse" should be defined or justified more clearly.*

Response: The manuscript now defines this term more carefully in Methods 2.5. It is retained as a descriptive regime label because it refers to a pattern in which affective intensity remains high while narrative scaffolding contracts toward the lower structural boundary of the space. The revised text makes explicit that this usage is structural and non-clinical.

Change: Methods 2.5 revised with a clarifying sentence on p. 8.

Attached Review: *Minor Comment 3. Point estimates for geometric extent and entropy should be accompanied by uncertainty reporting.*

Response: This has been addressed. The manuscript now reports bootstrap 95% confidence intervals for the human hull area and the contraction ratio, a two-sided permutation test for the between-agent comparison, and point estimates of occupancy entropy for both agents. The full inferential summary is reported in Supplementary Section S6.

Change: Results 3.5 revised on p. 14. Supplementary Section S6 expanded on pp. 3–4 of the Supplementary Information.

Attached Review: *Minor Comment 4. A summary table of the constructs and regimes would improve readability.*

Response: A summary table has been added near the beginning of the manuscript. It defines the three axes N, A, and D, and summarizes the four expressive regimes together with their operational interpretation.

Change: Table 1 added in the Introduction on p. 4.

Response to Reviewer #2

Inline Comments

Comment: *Statistical analysis: No.*

Response: In response to this concern, I re-examined the expressive-space geometry and corrected a structural issue in the original analysis. Because $D = N - A$ is a deterministic linear function of the two primary axes, three-dimensional convex-hull volume is not an appropriate measure of expressive extent on the exact data manifold. All geometric comparisons have therefore been recomputed as convex-hull area in the clipped (N', A') plane. I also expanded inferential reporting by adding bootstrap confidence intervals, a two-sided permutation test, occupancy entropy, and supplementary sensitivity analyses. The Pearson correlation $r(N, A) = 0.009$ is now reported directly in the main text in addition to the supplementary correlation table.

Change: Results 3.1 revised to report $r = 0.009$ on p. 11. Results 3.5 revised to report human and LLM convex-hull areas, contraction ratio, 95% confidence intervals, permutation test, and entropy values on p. 14. Supplementary Section S6 expanded on pp. 3–4 of the Supplementary Information. Supplementary sensitivity reporting added in S1 Text on p. 1 of the Supplementary Information.

Comment: *The study is well-structured and presents an interesting approach to understanding narrative–affect discrepancy.*

Response: Thank you for this assessment. No manuscript change was required in response to this comment.

Attached Comments: `CommentsPLOS.docx`

Reviewer #2 submitted an attached question file (`CommentsPLOS.docx`) containing nine explanatory prompts addressing the manuscript's core constructs (narrative–affect discrepancy $D = N - A$), the three-dimensional expressive geometry, the four expressive regimes, the ANAD dataset, the cost function J, correlation findings, convex hull measures, the aligned-model comparison, and methodological contributions.

These prompts are explanatory in character rather than point-by-point revision directives. I addressed their substance by strengthening the relevant sections of the manuscript: the Introduction now provides a clearer conceptual overview including a summary table (Table 1), the Methods sections (2.1–2.8) expand on dataset construction, operationalization, cost-function justification, and geometric rationale, the Results sections (3.1–3.5) add explicit statistical reporting, and the Discussion sections (4.1–4.5) deepen interpretive precision for aligned models, limitations of the affect measure, and methodological contributions. Supplementary Sections S1–S7 provide further detail on sensitivity, correlation structure, and inference. Together, these revisions ensure that each topic raised in the attached question file is addressed comprehensively within the revised manuscript.

Response to Reviewer #3

Comment: *The manuscript is exceptionally well-structured and technically sound. One suggestion: expand the discussion of the limitations of the affect measure (A), given that it is derived*

from lexicon-based sentiment scoring.

Response: A new paragraph has been added to the Limitations section to address this directly. The revised manuscript now states that A is derived from VADER, captures valence magnitude expressed in surface language, does not distinguish arousal from valence, and does not access suppressed or unexpressed affect. The revised text also makes clear that A should not be interpreted as a proxy for subjective felt intensity.

Change: Limitations 4.4 expanded with a paragraph on pp. 17–18.

Comment: *The near-zero correlation between N and A is interesting and the regime framework is novel.*

Response: Thank you for this assessment. The main text now reports the correlation explicitly in Results 3.1 and interprets its significance in Discussion 4.1. No additional change beyond this clarification was required.

Change: Results 3.1 clarified on p. 11. Discussion 4.1 clarified on p. 15.

Author-Initiated Correction Note

During the revision process, I re-ran the expressive-space analysis and identified a structural issue in the original geometric comparison. Because $D = N - A$ is a deterministic linear function of the two primary axes, observations lie on a plane in (N, A, D) space prior to clipping. The three-dimensional convex-hull volume reported in the original submission therefore reflected an artifact introduced by separate axis-wise clipping in transformed coordinates. In the revised manuscript, all geometric extent measures are computed as convex-hull area in the clipped (N', A') plane.

Updated results are as follows: Human convex-hull area = 99.514 (bootstrap mean; 95% CI $[98.596, 99.755]$); LLM convex-hull area = 58.679; contraction ratio = 1.696 (95% CI $[1.680, 1.700]$); permutation $p < 0.0001$ ($T = 10{,}000$); occupancy entropy Human = 4.654, LLM = 5.103. Under the corrected geometry, the aligned model occupies a substantially smaller empirical region of the human-derived expressive plane. Supplementary sensitivity analyses further show that the contraction ratio remains 1.70 after rounding across the reported threshold perturbation and rescaling conditions.

The revised manuscript also reports the final Pearson correlation between N and A directly in the main text as $r = 0.009$. This value is reproduced in the supplementary correlation table and is now integrated into the interpretation of the global expressive geometry.

The Data Availability statement now uses the Zenodo all-versions DOI (10.5281/zenodo.17632585) and explicitly identifies both the v1.1.0 release used for analysis and the current v1.3.0 release as alternative reproduction pathways. The bibliography was also updated to include the published *Data in Brief* article.

Summary of Reviewer-Driven and Author-Initiated Revisions

Item	Source	Location in revised manuscript
Definition of "regulated" clarified	Reviewer #1 (Attached MC1)	Introduction (p. 4); Discussion 4.1 (p. 15)
Cost function justification expanded	Reviewer #1 (Attached MC2)	Methods 2.6 (pp. 9–10)
Quantile-threshold robustness clarified	Reviewer #1 (Attached MC3)	Methods 2.5 (p. 8); S1 Text (p. 1 of SI)
"Collapse" label justified as descriptive	Reviewer #1 (Attached MC3/m2)	Methods 2.5 (p. 8)
LLM comparison limitations expanded	Reviewer #1 (Attached MC4)	Introduction (p. 3); Discussion 4.3 (p. 17); Limitations 4.4 (pp. 17–18)
Terminology unification (NCS)	Reviewer #1 (Attached m1)	Throughout; especially Introduction (pp. 3–4)
Bootstrap CI, permutation test, entropy added	Reviewer #2 / Reviewer #1 (Attached m3)	Results 3.5 (p. 14); S6 Text (pp. 3–4 of SI)
Overview table added	Reviewer #1 (Attached m4)	Table 1 in Introduction (p. 4)
Conclusion strengthened	Reviewer #1 (Inline)	Discussion 4.5 (pp. 18–19)
Transitions and proofreading improved	Reviewer #1 (Inline)	Introduction, Results, Discussion, throughout
VADER-based A limitation added	Reviewer #3	Limitations 4.4 (pp. 17–18)
Main-text reporting of $r(N, A) = 0.009$	Reviewer #2 / Reviewer #3	Results 3.1 (p. 11); Discussion 4.1 (p. 15)
3-D to 2-D geometric correction	Author reanalysis	Abstract (p. 1); Methods 2.1 and 2.7 (pp. 6, 10); Results 3.5 (p. 14); Discussion 4.2 (pp. 16–17); S6 Text (pp. 3–4 of SI)
Expanded inferential reporting	Author reanalysis	Results 3.1 and 3.5 (pp. 11, 14); S1 Text; S6 Text
Code Availability statement added	Journal Requirement	Code Availability section (p. 19)
Data Availability updated (all-versions DOI; v1.1.0 and v1.3.0 identified)	Author revision	Data Availability (p. 19)

Reference and terminology harmonization	Author revision	References; throughout

I hope these revisions adequately address the concerns raised and improve the manuscript's clarity, rigor, and reproducibility.

Respectfully,

Ryan SangBaek Kim, Ph.D.
Ryan Research Institute (RRI)
Paris, France
ryan@ryanresearch.org
