# Supplementary material for: Narrative–affect discrepancy as a regulated degree of freedom in 351,734 relationship narratives
Source: PLoS One. 2026 May 12;21(5):e0348715. doi: 10.1371/journal.pone.0348715 (PMC13166951; doi:10.1371/journal.pone.0348715)
Supplement: Attachment — Submitted filename: COMMENTS.docx [file pone.0348715.s011.docx]

This publication uses an extraordinarily large dataset of 351,734 relationship narratives to propose a fresh and ambitious large-scale analysis of narrative–affect discrepancy. The main contribution is novel and has the potential to influence computational social science, narrative psychology, and affective computing by understanding discrepancy (D = N − A) as an organized and regulated dimension instead of noise. The study is clearly positioned against popular beliefs that coherence between affect and narrative is normative in this well-motivated introduction. Particularly persuasive is the move toward a geometric and population-level framework. In general, the process is transparent and rigorous. It is reasonable to utilize a two-dimensional expressive space (N, A) with derived discrepancy, and the geometric analysis's validity is strengthened by the conversion from 3D volume to 2D convex hull area. Robustness is improved with the addition of clipping, bootstrapping, and permutation testing, and the endeavour to achieve repeatability through shared datasets, code, and thorough procedural descriptions is praiseworthy. Sensitivity analyses boost confidence in the stability of results, and the regime classification is both empirically supported and intuitive. Nonetheless, there are a few points that could use more explanation or clarity. First, although useful, VADER's operationalization of affective intensity is still a drawback. Although noted, the publication should go into greater detail about how this decision could skew the results, especially when it comes to identifying nuanced or context-dependent emotional expression. Second, in order to prevent overstretching into psychological statements about individual behaviour, the interpretation of "regulation" at the population level should be approached with ongoing vigilance. Although this warning is provided by the authors, it would be beneficial to reiterate it in the consequences discussion. Although the comparison with the RLHF-aligned language model is intriguing, it should be understood as experimental. Strong causal assertions are wisely avoided in the text, but this section would benefit from more discussion of alternate possibilities (such as prompt design, decoding restrictions, or dataset mismatch). Future research could overcome this restriction by incorporating comparisons with various models or combinations. Given the use of de-identified, derived features and publicly available data, the study seems to comply with data privacy standards from an ethical standpoint. Conflicts of interest, dual publication, and research ethics are not now a problem. Strong remarks on data availability and transparency are in line with open science principles. To sum up, this is a rigorous and methodologically sound contribution that offers a useful framework for examining how emotions are expressed in stories. The work is ready for publication and should encourage more research in this field with a few minor clarifications and a more thorough assessment of its limits.
