# Supplementary material for: Narrative–affect discrepancy as a regulated degree of freedom in 351,734 relationship narratives
Source: PLoS One. 2026 May 12;21(5):e0348715. doi: 10.1371/journal.pone.0348715 (PMC13166951; doi:10.1371/journal.pone.0348715)
Supplement: Attachment — Submitted filename: CommentsPLOS.docx [file pone.0348715.s008.docx]

Comments:

1. Explain the concept of Narrative–Affect Discrepancy (D = N − A) introduced in the paper. How does this framework challenge traditional assumptions in psychology and affective computing about emotional coherence?
2. Describe the three-dimensional expressive space defined by:

- Narrative complexity (N)
- Affective intensity (A)
- Signed discrepancy (D)

How does this geometric representation help in understanding emotional regulation at scale?

3. The study identifies four expressive regimes:

- Coupled expression
- Strategic understatement
- Strategic overstatement
- Collapse

Explain each regime in detail with reference to its structural characteristics and psychological interpretation.

4. Discuss the ANEST Narrative–Affect Dataset (ANAD v1.1.0).
   How were N and A operationalized, and why is the use of derived features (instead of raw text) important for ethical and methodological reasons?
5. Explain the data-driven cost function J(N′,A′,D′)J(N′, A′, D′)J(N′,A′,D′) developed in the study.
   How does this model conceptualize expressive regulation as a trade-off between exposure risk and cognitive effort rather than discrepancy minimization?
6. Analyze the correlation findings reported in the paper.
   Why is the near-independence between narrative complexity (N) and affective intensity (A) significant for the study’s main claims?
7. What is meant by convex hull volume in the clipped (N′, A′, D′) space?
   Explain how expressive volume and occupancy entropy are used to quantify expressive freedom in humans.
8. The study compares human narratives with an RLHF-aligned GPT-4-class language model.
   Discuss the findings related to expressive contraction (approximately 4.5× smaller hull volume).
   What does this imply about alignment constraints in large language models?
9. Explain how the study contributes methodologically to:

- Narrative psychology
- Computational social science
- AI alignment research

Why is the “measurement-first” approach emphasized?
