# Supplementary material for: Narrative–affect discrepancy as a regulated degree of freedom in 351,734 relationship narratives
Source: PLoS One. 2026 May 12;21(5):e0348715. doi: 10.1371/journal.pone.0348715 (PMC13166951; doi:10.1371/journal.pone.0348715)
Supplement: Attachment — Submitted filename: Review Comments.docx [file pone.0348715.s009.docx]

**Review Comments**

**Manuscript ID:** PONE-D-26-02582
**Title:** *Narrative–Affect Discrepancy as a Regulated Degree of Freedom in 351,734 Relationship Narratives*

**Summary**

A comprehensive, methodologically transparent investigation of narrative–affect discrepancy in more than 350,000 relationship narratives is presented in this publication. Instead of seeing the disparity between narrative complexity and conveyed affect as residual noise, the author presents a geometric framework that views it as an organized and controlled expressive dimension. The study provides a novel descriptive lens on emotional expression and expressive restrictions using quantile-based regime definitions, a data-anchored cost landscape, and a comparison projection of an aligned language model. The study is well-executed, ambitious, and compliant with PLOS ONE's evaluation standards and scope. The analysis workflow is replicable, the dataset is sizable, and the presented results mostly corroborate the assertions. After making minor edits that mostly focus on conceptual clarity and interpretive bounds, I think the manuscript is ready for publishing.

**Major Comments**

**1. Clarification of the Term “Regulated”**

Narrative–affect discrepancy is frequently described in the manuscript as a "regulated degree of freedom." Although the population-level pattern is persuasively shown, readers may understand the word "regulated" as suggesting psychological control or optimization at the individual level.

**Recommendation:**

Please make it clear that "regulation" refers to persistent constraint patterns that can be seen in aggregate data rather than to conscious or mechanical regulation by people. It is used in a descriptive, population-level sense. In the Introduction or Discussion, a brief paragraph providing clarification would aid in avoiding misunderstandings.

**2. Justification and Interpretation of the Cost Function**

- Elegant and essential to the manuscript's interpretive framework is the suggested cost landscape (Section 2.6).
- However, a more thorough explanation would be helpful for a number of modeling decisions:

• The use of quadratic sanctions
• Rather than centering deviation at zero, do so around the median discrepancy (D*).
• Adding a word for deviation while claiming that the discrepancy is not minimized

**Recommendation:**

Kindly provide a succinct explanation of these design decisions and make it clear that the cost function is an abductive, illustrative function rather than a fitted or inferred objective function. By making this explanation, the methodological transparency would be strengthened and the cost model would not be interpreted as normative.

**3. Dependence on Quantile-Based Regime Thresholds**

Empirical quantiles are used to determine the four expressive regimes, which makes sense but is a little arbitrary. Although the supporting information contains sensitivity analyses, this dependence may be acknowledged more explicitly in the main text.

**Recommendation:**

In the Methods or Results section, include a succinct sentence stating:

• Both the qualitative interpretation and the regime structure withstand reasonable adjustments to quantile thresholds.
• "Coupled expression" is not a theoretically privileged state; rather, it is a residual category by design.

**4. Interpretation of the Language Model Comparison**

Although the comparison study using an RLHF-aligned language model is intriguing and skilfully done, there is a chance that readers would mistakenly ascribe the observed expressive contraction to alignment goals.

**Recommendation:**

Please emphasize that in order to further attenuate causal language:

• One aligned model under a particular prompt and decoding configuration is used for the comparison.
• Rather than only alignment, expressive contraction can also be a result of safety filtering, prompting limits, or training distribution.

By doing this, causal overreach would be avoided and the descriptive framing would be strengthened.

**Minor Comments**

1. Consistency in Terminology: "Narrative–Control Space," "NCS," and "NADI control space" are used interchangeably throughout the article. Please introduce alternatives only once parenthetically and standardize nomenclature.
2. Labelling of the "Collapse" Regime: Although the non-clinical objective is made obvious, it would be better to provide a brief explanation for the term "collapse" (as opposed to "compression" or a similar term).
3. Statistical Reporting: When possible, include point estimates for convex hull volume and entropy comparisons with bootstrap ranges or confidence intervals.
4. 4. Readability: The Introduction is complex but intellectually rich. Accessibility might be increased by including a quick graphic or table at the beginning of the work that summarizes N, A, D, and the four regimes.

**Ethical and Data Considerations**

De-identification, avoiding verbatim content, and transparent data availability are only a few of the ethical issues that the manuscript exemplifies. There are no issues in this area.

**Recommendation to the Editor**

**Recommendation:** **Accept with Moderate Revisions**

The work is repeatable, adheres strictly to PLOS ONE's guidelines, and is methodologically sound. The paper will be strengthened and made more accessible to a wide range of interdisciplinary readers by addressing the aforementioned problems, particularly explanations of interpretation and modelling decisions.
